# Plant Invasion Ecology

**DOI:** 10.3390/plants12223887

**Published:** 2023-11-17

**Authors:** Alexander P. Sukhorukov

**Affiliations:** 1 Department of Higher Plants, Biological Faculty, Lomonosov Moscow State University, 119234 Moscow, Russia; suchor@mail.ru; Tel.: +7-(495)-939-1827; 2 Laboratory Herbarium (TK), Tomsk State University, Lenin Ave. 36, 634050 Tomsk, Russia

This article belongs to the Special Issue “Plant Invasion Ecology”.

The number of naturalized and invasive plants has increased dramatically all over the world in the past several decades. Invasive alien plant species convey their invasibility to successful invasion through pre-adaptation and post-introduction rapid adaptive evolution. Many invasive species can negatively alter natural ecosystems (e.g., meadows, steppes, or forests) and often form monodominant plant communities. They are among the leading threats to native wildlife. In many cases, the invasive mechanism and ecological consequences of species naturalization are still poorly understood and difficult to predict.

The tasks and problems elucidated by the authors within this topic can be divided into several main categories: (1) genetic, physiological, and morphological traits as the main factors influencing invasive potential of a species; (2) the application and development of different methods for studying plant invasions, including interdisciplinary approaches; (3) interactions between native and non-native plant species, including changes in taxonomic diversity of communities; and (4) a detailed investigation of an escape of a cultivated and ornamental species to the wild.

Jiang et al. (2023) [1] reviewed articles about genetic diversity of invasive alien species in and out of their native habitats and stated that a general reduction in genetic diversity has been found more often in non-native populations than in native ones. Nevertheless, those authors concluded that a change in genetic diversity has no substantial effect on the outcome of an invasion process. A representative cytogeographical investigation was conducted about invasive *Solidago canadensis* (Asteraceae) [2], with subsequent insight into the invasive mechanisms and morphological variations driven by genetic factors. Tian et al. [2] showed for the first time the presence of polyploids in European populations of this species. They also propose considering *S. canadensis* in a broad sense including *S. altissima*. Tesfay et al. [3] examined plasticity toward water stress in native and invasive populations of *Opuntia ficus*-*indica* (a prevalent invader in arid and semiarid ecosystems) and found that invasive populations manifest enhanced phenotypic plasticity in response to water availability, and this characteristic contributes to their colonizing potential. Several articles within the topic deal with reproductive traits, which are known to play a substantial role in the spread and establishment of alien taxa [4]. Potential fecundity was studied in *Cytisus scoparius* (Fabaceae) within its native and alien ranges [5], while seed germination under different conditions was investigated for *Iris pseudacorus* (Iridaceae) [6], *Prosopis juliflora* [7], and *Acacia mearnsii* [8] (both Fabaceae).

Predictive modeling of the invasiveness and potential geographical distributions was developed for several widespread vascular plants: *Pueraria montana* (Fabaceae) [9] and *Impatiens glandulifera* (Balsaminaceae) [10] in North America and *Tagetes minuta* [11], *Ageratina adenophora* [12], and *Ambrosia artemisiifolia* [13] (all Asteraceae) in China. These models allow habitat suitability to be calculated for the invasive taxa and long-term projections of invasions to be made, thus helping to monitor and manage their spread in areas of concern.

Another focus of research on invasive plant taxa is on their interaction with native species in colonized habitats. Conte et al. [14] analyzed the competition between native seagrass *Cymodocea nodosa* (Cymodoceaceae) and alien *Halophila stipulacea* (Hydrocharitaceae) in the Aegean Sea and found that *C. nodosa* and its epiphytic bacterial communities are negatively affected by the presence of exotic *H. stipulacea*. An in-depth analysis was also conducted regarding the impact of aggressive invader *Prosopis velutina* on the native- profile and diversity of woody plants in South Africa [15] and regarding interactions between two alien species (*Alliaria petiolata* and *Hesperis matronalis*; both Brassicaceae) and a native species (*Ageratina altissima*; Asteraceae) in North America [16].

The status of alien *Kalanchoe* taxa (Crassulaceae) was reviewed in Ecuador, where the genus is widely used as an ornamental plant [17]. Five species, including one nothospecies, were categorized as invasive. Domestication of some *Kalanchoe* taxa may also affect rich endemic flora of the Galapagos Archipelago. 

In this editorial, I have mentioned only some of the papers published in this Special Issue. In total, 20 articles were published on the title topic. Notably, many of them give special attention to the influence of climate change and global warming on invasion dynamics of the plant species under study. 
